# Muscle-Specific Modulation of Spinal Reflexes in Lower-Limb Muscles during Action Observation with and without Motor Imagery of Walking

**DOI:** 10.3390/brainsci9120333

**Published:** 2019-11-21

**Authors:** Naotsugu Kaneko, Yohei Masugi, Noboru Usuda, Hikaru Yokoyama, Kimitaka Nakazawa

**Affiliations:** 1 Department of Life Sciences, Graduate School of Arts and Sciences, The University of Tokyo, Komaba, Meguro-ku, Tokyo 153-8902, Japan; kaneko-naotsugu526@g.ecc.u-tokyo.ac.jp (N.K.); ymasugi@tiu.ac.jp (Y.M.); n-usuda@g.ecc.u-tokyo.ac.jp (N.U.); 2 Japan Society for the Promotion of Science, Chiyoda-ku, Tokyo 102-0083, Japan; hyokoyama@m2.tuat.ac.jp; 3 Institute of Sports Medicine and Science, Tokyo International University, Matoba, Kawagoe-shi, Saitama 350-1198, Japan; 4 Department of Electrical and Electronic Engineering, Tokyo University of Agriculture and Technology, Tokyo 184-8588, Japan; 5 Rehabilitation Engineering Laboratory, Toronto Rehabilitation Institute, University Health Network, Toronto, ON M4G 3V9, Canada

**Keywords:** action observation, motor imagery, walking, spinal reflex, transcutaneous spinal cord stimulation

## Abstract

Action observation (AO) and motor imagery (MI) are useful techniques in neurorehabilitation. Previous studies have reported that AO and MI facilitate corticospinal excitability only in those muscles that are active when actually performing the observed or imagined movements. However, it remained unclear whether spinal reflexes modulate multiple muscles simultaneously. The present study focused on AO and MI of walking and aimed to clarify their effects on spinal reflexes in lower-limb muscles that are recruited during actual walking. Ten healthy males participated in the present study. Spinal reflex parameters evoked by transcutaneous spinal cord stimulation were measured from five lower-limb muscles during rest, AO, and AO combined with MI (AO + MI) conditions. Our results showed that spinal reflexes were increased in the tibialis anterior and biceps femoris muscles during AO and in the tibialis anterior, soleus, and medial gastrocnemius muscles during AO + MI, compared with resting condition. Spinal reflex parameters in the vastus medialis muscle were unchanged. These results indicate the muscle-specific modulations of spinal reflexes during AO and AO + MI. These findings reveal the underlying neural activities induced by AO, MI, and their combined processes.

## 1. Introduction

Action observation (AO) and motor imagery (MI) are useful rehabilitation techniques for patients with neurological disorders. In humans, neural systems match AO, MI, and action execution. Both AO and MI are known to modulate those neural systems that relate to observed and imagined movements without action execution and muscle contraction. Several recent studies have reported that rehabilitation involving AO and MI could facilitate recovery of motor functions after neurological disorders such as stroke and Parkinson’s disease [1,2,3,4]. Although the neural systems related to AO and MI are not fully understood, previous studies using electrophysiological techniques in healthy people have reported effects of AO and/or MI on excitability changes in the central nervous system [5,6,7,8,9]. For example, Fadiga et al. [7,8] reported that both AO and MI facilitate the excitability of corticospinal tracts which can be assessed through motor-evoked potentials (MEPs) induced by transcranial magnetic stimulation (TMS) of the primary motor cortex. Fadiga et al. also investigated electromyographic (EMG) activities during object grasping and arm elevation, as well as MEP modulations during AO of the same movements in hand and arm muscles. They showed that MEPs were increased during AO of grasping movements or arm elevation only in those muscles that are active during the corresponding actual movement [7]. Furthermore, Fadiga et al. reported that during MI of forearm flexion, MEPs in the biceps brachii muscle were increased while those in the opponens pollicis muscle, which is not recruited during forearm flexion, were unchanged [8]. These studies showed that MEPs during AO and MI are increased only in muscles which are active when actually performing observed or imagined movements.

AO and MI affect not only the excitability of the corticospinal tract but also of spinal reflex circuits. Previous studies have shown that AO and MI facilitate the excitability of spinal reflex circuits which can be assessed using the Hoffmann reflex (H-reflex) [5,6,9]. However, only a few studies examined spinal reflexes in multiple muscles during AO and MI. Thus, it is still unclear whether spinal reflexes are modulated by observed and imagined movements similar to the modulation of corticospinal excitability. Moreover, most of the previous studies have focused on AO and MI of single-joint movements controlled by a few muscles. The present study focused on AO and MI of walking that is a complex whole-body movement controlled by many muscles. Our studies recently have examined the neural mechanism underlying AO and MI of walking, and specifically AO combined with MI (AO + MI). This combination makes the mental simulation clearer than MI alone; previous studies reported that AO + MI increased MEP amplitudes over those in either AO or MI alone [10,11]. Furthermore, our studies showed that AO + MI of walking increased both MEPs in the tibialis anterior (TA) and soleus (SOL) muscles [12], as well as SOL H-reflex amplitudes [13]. These results showed that AO + MI of walking facilitates not only the excitability of the corticospinal tract but also that of spinal reflex circuits, suggesting facilitatory effects of AO + MI to both the primary motor cortex and the spinal motoneurons. Also, the MEP increase in both TA and SOL muscles suggested that AO + MI simultaneously facilitated corticospinal excitability in the muscles related to walking [12]. However, since the H-reflex was only assessed in the SOL muscle in our previous study [13], it was unknown whether the excitability of spinal reflex circuits would be modulated in the other lower-limb muscles during AO and MI of walking.

The research question in the present study is, therefore, whether AO and MI increase the excitability of spinal reflexes in muscles recruited during actual walking in the same way as they modulate corticospinal excitability. The basic activation patterns of lower-limb muscles during walking are considered to be generated by spinal central pattern generators [14,15]. Therefore, our special attention was focused on the question: Do AO and MI of walking facilitate spinal reflexes in all lower-limb muscles regardless of the walking phase or in each muscle according to the walking phase in which this particular muscle is active while walking (e.g., the TA and SOL muscles in the swing and stance phases, respectively)?

Transcutaneous spinal cord stimulation (tSCS) is one of the techniques to answer this question. The technique can simultaneously evoke spinal reflex responses in multiple lower-limb muscles [16,17]. Measurement of the spinal reflexes in multiple muscles is expected to extend the knowledge of the neural mechanisms involved in AO and MI of walking. Therefore, the purpose of the present study is to investigate using tSCS the effects of AO and AO + MI of walking on spinal reflexes of lower-limb muscles recruited during actual walking and to examine whether AO and AO + MI modulate spinal reflex excitability in the same way as they modulate corticospinal excitability. The study results may aid in the neurorehabilitation of patients with neurological gait disorders.

## 2. Materials and Methods

### 2.1. Participants

Ten healthy males aged 22–32 years (mean ± standard deviation, 26.2 ± 3.3 years) with no history of neurological disorders participated in the present study after providing informed consent. All experimental procedures were approved by the local ethics committee of the University of Tokyo (533-2). This study was performed in accordance with the Declaration of Helsinki.

### 2.2. Electromyographic Recordings

EMG recordings were made using bipolar Ag/AgCl surface electrodes (Vitrode F-150S; Nihon Kohden, Tokyo, Japan) on the TA, SOL, medial gastrocnemius (MG), vastus medialis (VM), and biceps femoris long head (BF) muscles. After cleaning the skin with alcohol, the electrodes were placed over the muscle belly with an interelectrode distance of 20 mm. The EMG signals were amplified (×1000) and filtered with a band-pass filter between 15 Hz and 3 kHz using a bio-amplifier system (MEG-6108; Nihon Kohden, Tokyo, Japan).

### 2.3. Transcutaneous Spinal Cord Stimulation

Participants were asked to maintain the supine position during the tSCS experiments. To evoke spinal reflexes of multiple muscles in the lower limbs, a constant-current electrical stimulator (DS7A, Digitimer Ltd., Welwyn Garden City, Hertfordshire, UK) was set to a pulse width of 1 ms. The anode (100 × 75 mm) was placed over the abdomen, and the cathode (50 × 50 mm) was placed on the skin on the midline between the spinous processes of the higher lumbar vertebrae [18]. Prior to the experiment, the location—where a single pulse stimulation produced the largest response of the lower-limb muscles—was selected (T12/L1 *n* = 3, L1/L2 *n* = 6, L2/L3 *n* = 1). Then, the recruitment curve of the tSCS-evoked responses was obtained to determine the stimulus intensity in each participant [19]. To avoid ceiling and floor effects of the response size, the stimulus intensity was set to obtain tSCS-evoked responses on the ascending limb of the recruitment curve. To confirm that all tSCS-evoked responses were caused by the activation of afferent fibers, a double-pulse stimulation (50 ms interval) was applied at the beginning of each experiment [16,17].

### 2.4. Experimental Protocols

Participants were asked to look at a mirror reflecting a display during this experiment. To prevent movement of the tested leg, the right ankle joint was fixed at 10 degrees plantar flexion using an ankle-foot orthosis. Both video presentation and stimulation timing were controlled using a custom LabVIEW program (National Instruments Inc., Austin, TX, USA). Two 7-s videos with a frame rate of 30 fps—one portraying walking and the other displaying a fixation cross—were used. The former video showed a man walking on the floor for 10 steps.

**Figure 1:**
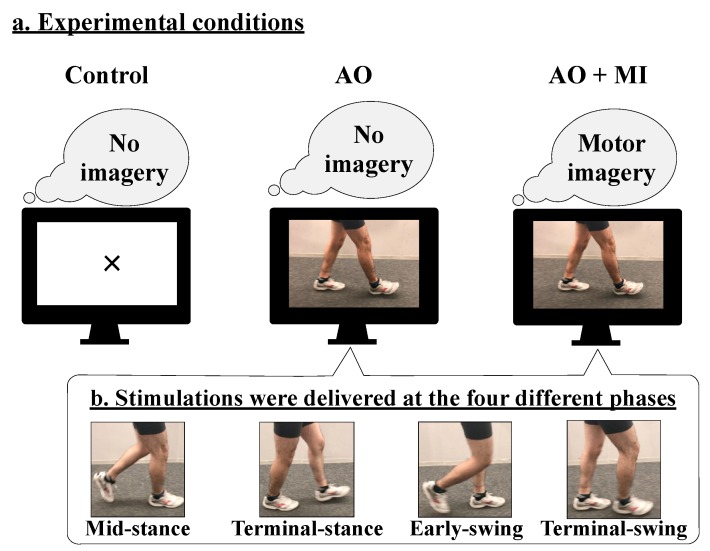
Experimental setup. (**a**) Spinal reflex parameters were determined for the following three conditions: control, action observation (AO), and AO with motor imagery (AO + MI). (**b**) In the AO and AO + MI conditions, electrical stimulation was randomly delivered during the following four phases: mid-stance, terminal-stance, early-swing, and terminal-swing.

This study investigated the following three conditions: (1) control, (2) AO, and (3) AO + MI (Figure 1a). In the control condition, the participants were asked to look at the center of the fixation cross that was presented at the center of the monitor. In the AO condition, the walking video was presented on the monitor, and the participants were asked to observe the man’s legs and not to imagine anything else. In the AO + MI condition, the same walking video was presented on the monitor, and the participants were asked to observe the man’s legs and to imagine that they were walking similar to the man in the movie. In all conditions, the participants were given the same instructions. The participants practiced the tasks of the AO and AO + MI conditions before measurements. They were asked to relax their bodies and concentrate on each task during the recordings.

During walking, the H-reflex amplitude is modulated in a phase-dependent manner [20]. To examine whether a phase-dependent modulation of spinal reflexes occurs in AO and AO + MI conditions similar to that during actual walking, electrical stimulation was applied at each of the following walking phases: (1) mid-stance, (2) terminal-stance, (3) early-swing, and (4) terminal-swing of the right leg (at 2883, 3167, 3533, and 3767 ms after video onset, respectively; Figure 1b). Since the order of the stimulation timing was randomized across the trials using the LabVIEW program, the participants could not anticipate the timing of the stimulation. A total of three sets were performed. One set consisted of the control, AO, and AO + MI conditions conducted in random order. A break of at least 2 min was provided between two sets, to prevent a decrease in concentration. For each condition of one set, 12 trials were performed. In each trial, one response of the tSCS-evoked spinal reflex was recorded in the TA, SOL, MG, VM, and BF muscles. Thus, for each participant, 36 responses (3 sets × 12 stimuli) were recorded for control, AO, and AO + MI conditions in each muscle. At the end of each experiment, the participants were asked to contract the muscles as strongly as possible against manual resistance and hold it for 3 seconds. The EMG signals were recorded when the participants performed the maximum voluntary contraction (MVC) for all the recorded muscles.

### 2.5. Data and Statistical Analyses

The signals from 100 ms before to 200 ms after the stimulus were sampled at 10 kHz using a 16-bit A/D converter (NI USB-6259 BNC; National Instruments Inc.). The peak-to-peak amplitudes (mV) of tSCS-evoked spinal reflexes in the TA, SOL, MG, VM, and BF muscles were calculated off-line. For each participant, the amplitudes (mV) were averaged in each of the control, AO, and AO + MI conditions. The mean amplitudes of the tSCS-evoked spinal reflexes during AO and AO + MI were normalized as the percentage of the mean amplitudes in the control conditions. The normalized amplitudes (% control) in the AO and AO + MI conditions were averaged in each condition and each phase. The background EMG activity in the TA, SOL, MG, VM, and BF muscles was calculated as the root mean square of the EMG signals during the 50 ms that preceded the stimuli and was normalized with that during the MVC. 

A paired *t*-test was conducted to compare the mean amplitudes of the responses induced by the first stimulus to those by the second stimulus in each muscle.

For the tSCS-evoked spinal reflex amplitudes in each muscle, one-sample t-tests were conducted to compare the control condition (100%) with the AO or AO + MI conditions. For the amplitudes (% control) and background EMG activities (% MVC), statistically significant differences were evaluated using two-way repeated-measures analysis of variance (rm-ANOVA; two conditions (AO and AO + MI), four phases (mid-stance, terminal-stance, early-swing, and terminal swing)) and one-way rm-ANOVA (four phases (mid-stance, terminal-stance, early-swing, and terminal swing)) for each condition (AO and AO + MI). If the rm-ANOVA tests showed a significant main effect, multiple comparisons were performed using the post-hoc test. If the two-way rm-ANOVA tests showed a significant interaction, simple main effect tests were conducted to examine the source of the interaction.

A correlation analysis (Pearson’s test) was performed to test the relationships of modulation between the lower-limb muscles in each condition.

The significance level was set at *p* < 0.05 in all statistical tests. Cohen’s d and partial eta squared (η_p_^2^) values were calculated as effect size (ES) indices for the one-sample *t*-test, paired *t*-test, rm-ANOVA, and post-hoc test. All *p*-values were uncorrected for multiple comparisons. We confirmed normal distribution and homogeneity of variances of each variable before the *t*-tests and rm-ANOVA tests using the Shapiro–Wilk test and Mauchly’s test of sphericity. Data were described as the mean ± standard error of measurement (SEM).

## 3. Results

**Figure 2:**
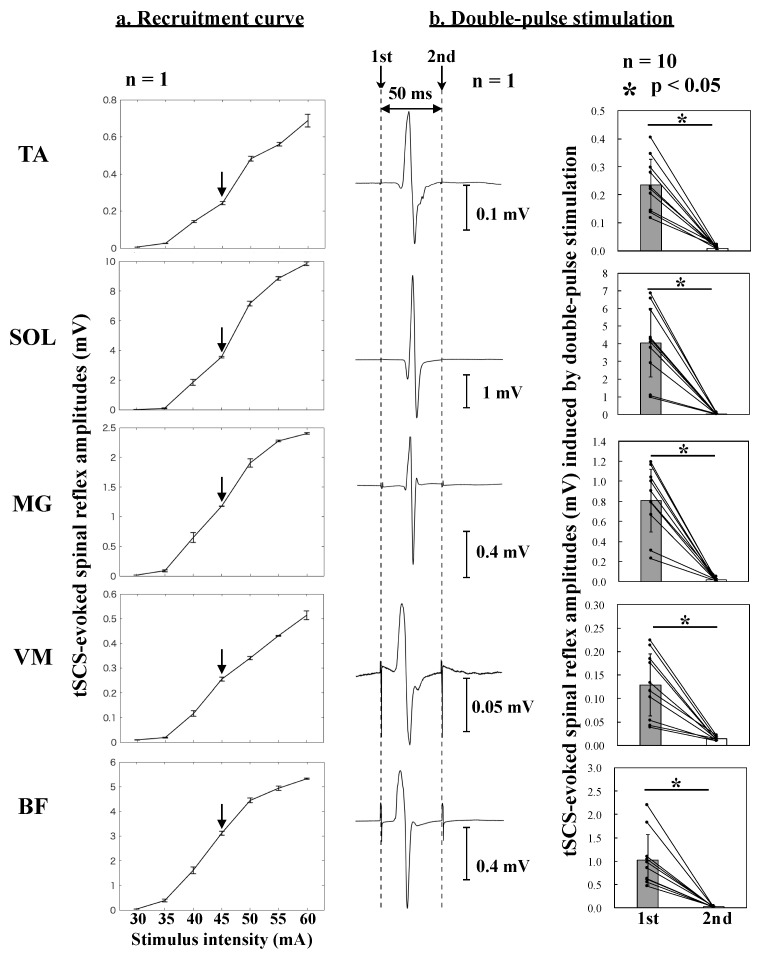
Recruitment curve and double-pulse stimulation. (**a**) Typical recruitment curve recordings of transcutaneous spinal cord stimulation (tSCS)-evoked spinal reflexes obtained in a single participant. Arrows indicate the stimulus intensity on the ascending limbs of each recruitment curve. This intensity was used in subsequent experiments to measure tSCS-evoked spinal reflexes in all muscles. (**b**) Representative examples (left) of averaged tSCS-evoked spinal reflex responses to the first and second stimulations obtained in a single participant. Average tSCS-evoked spinal reflex amplitudes (right, *n* = 10) of the responses to the first (grey bar) and second stimulations (white bar). Error bars represent standard errors of measurement. Each circle represents an individual data point. * Significant difference between the first and the second response (*p* < 0.05, effect size d > 1).

Figure 2a shows the recruitment curves of the tSCS-evoked spinal reflexes obtained from an individual participant. Based on the results of these recruitment curves, the stimulus intensity was set to obtain tSCS-evoked spinal reflexes on the ascending limbs of the recruitment curves. Figure 2b displays the first and second responses evoked by the first and second stimulation, respectively. The peak-to-peak amplitudes of the second response were significantly lower than those of the first response in all recorded muscles (*p* < 0.05, d > 1, paired *t*-test). 

The average tSCS-evoked spinal reflex amplitudes with SEMs in the control conditions in the TA, SOL, MG, VM, and BF muscles were 0.254 ± 0.023 mV, 4.30 ± 0.69 mV, 0.900 ± 0.108 mV, 0.143 ± 0.024 mV, and 1.20 ± 0.18 mV, respectively. For the tSCS-evoked spinal reflex amplitudes normalized to those under control conditions, the one-sample *t*-test showed that the amplitudes of the SOL muscle during the AO + MI condition were significantly greater than those during the control condition regardless of the walking phase (*p* < 0.05, d = 0.991, Table 1). There were tendencies to increased amplitudes during the AO condition in TA and BF muscles, as well as during the AO + MI condition in TA and MG muscles (Table 1).

**Figure 3:**
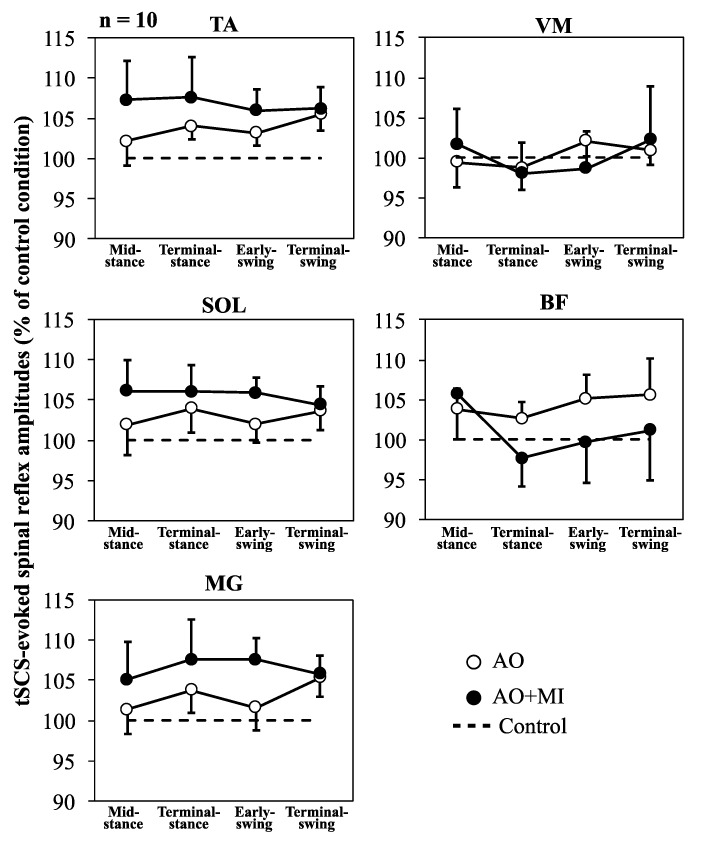
Mean amplitudes (*n* = 10) of normalized transcutaneous spinal cord stimulation (tSCS)-evoked spinal reflexes in the tibialis anterior (TA), soleus (SOL), medial gastrocnemius (MG), vastus medialis (VM), and biceps femoris long head (BF) muscles in action observation (AO; open circles) and AO with motor imagery (AO + MI; closed circles) conditions. Error bars represent standard errors of measurement. Dashed lines (100%) indicate baseline value (100%; i.e., tSCS-evoked spinal reflex amplitude under control conditions). There were no significant main effects regarding condition, phase, or their interaction in any of the recorded muscles.

Figure 3 shows the mean amplitudes of the spinal reflexes (% of control) with SEM for AO and AO + MI conditions relative to control (100%). For the amplitudes normalized to control conditions, the two-way rm-ANOVA test (condition × phase) did not reveal significant main effects involving condition, phase, or their interaction in any of the recorded muscles (Table 2). Also, the one-way rm-ANOVA test (phase) did not reveal a significant main effect of phase in any recorded muscle (Table 2).

The mean background EMGs (% MVC) of TA, SOL, MG, VM, and BF muscles under all conditions were 0.32 ± 0.07, 0.78 ± 0.11, 0.82 ± 0.14, 0.96 ± 0.15, and 1.17 ± 0.07 (% MVC), respectively. The two-way rm-ANOVA did not show significant main effects or interactions of the background EMG for any muscle. Similarly, the one-way rm-ANOVA did not show a significant main effect for the background EMG of any muscle in any condition.

**Figure 4:**
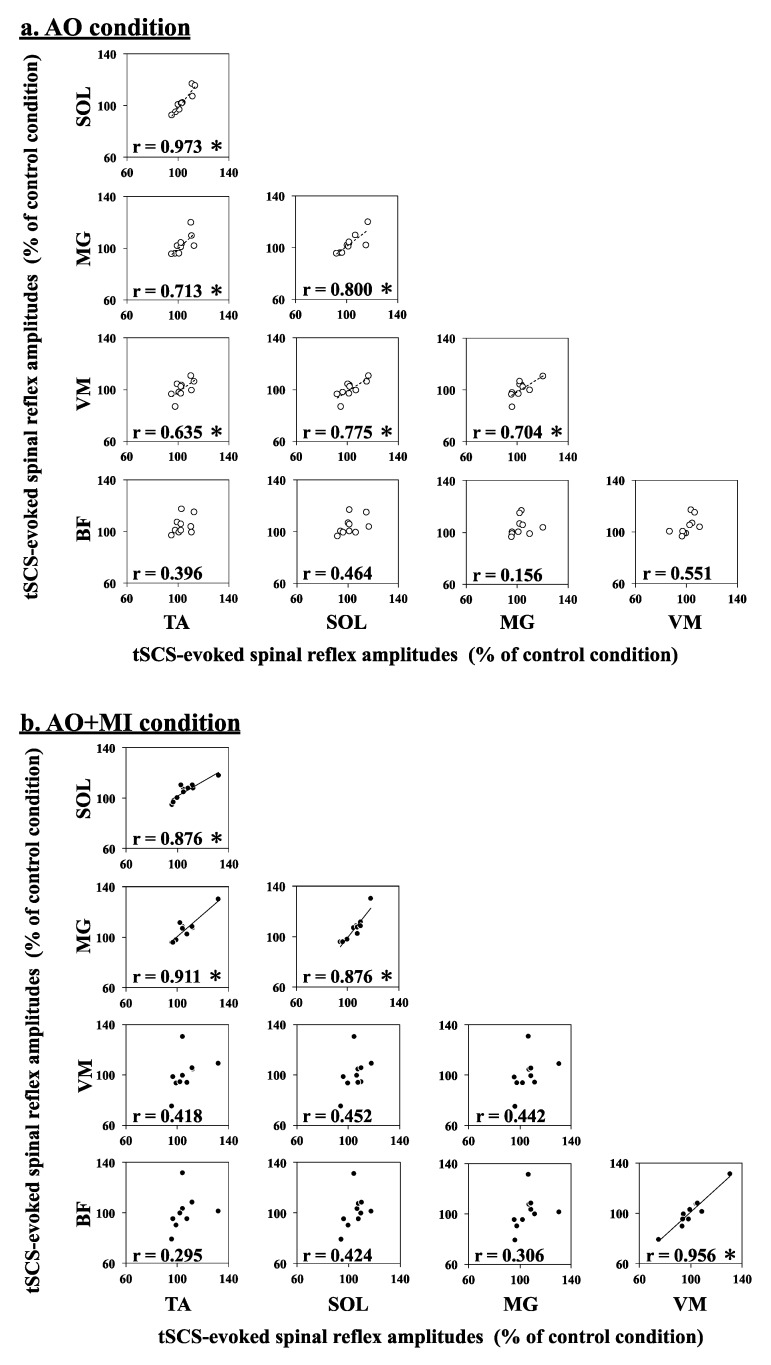
Pearson correlation of simultaneous modulation patterns of transcutaneous spinal cord stimulation (tSCS)-evoked spinal reflexes in different lower-limb muscles during action observation (AO; open circles; (**a**)) and AO with motor imagery (AO + MI; closed circles; (**b**)) conditions. Each plot indicates the mean tSCS-evoked spinal reflexes (% of control) in each muscle from all participants. The r values indicate Pearson’s correlation coefficients. * Significant correlation between two lower-limb muscles (*p* < 0.05).

Figure 4 displays the Pearson correlation of simultaneous modulation patterns of tSCS-evoked spinal reflexes between each lower-limb muscle during AO and AO + MI. These correlations indicate which muscles are simultaneously modulated during AO and AO + MI. The Pearson correlation analysis revealed significant strong positive correlations of tSCS-evoked spinal reflex amplitudes (% of control) between each of the TA, SOL, MG, and VM muscles in the AO condition. Moreover, significant positive correlations between each of the TA, SOL, and MG muscles, as well as between the VM and BF muscles, were observed in the AO + MI condition (for more details, see Table 3). Thus, the modulation patterns of spinal reflex excitability were similar between the TA, SOL, MG, and VM muscles during AO and between the TA, SOL, and MG muscles and between the VM and BF muscles during AO + MI.

## 4. Discussion

Using tSCS, the present study examined the modulation of spinal reflexes in the lower-limb muscles during AO and AO + MI of walking. The results of the present study showed that tSCS-evoked spinal reflexes were increased in the TA and BF muscles during AO (TA: *p* < 0.1, d = 0.827; BF: *p* < 0.1, d = 0.825) and in the SOL, TA, and MG muscles during AO + MI (SOL: *p* < 0.05, d = 0.991; TA: *p* < 0.1, d = 0.844; MG: *p* < 0.1, d = 0.842), regardless of the observed walking phase (Table 1, Figure 3). These results indicate muscle-specific facilitation of lower-limb spinal reflexes and a difference in their modulation during AO and AO + MI. In the following discussion sections, we suggest that the muscle-specific facilitation of spinal reflexes is related to the connectivity strength between the brain and the muscles.

### 4.1. Muscle-Specific Facilitation of Spinal Reflexes in Lower-Leg Muscles during AO + MI

Previous studies using TMS reported that AO and MI facilitate corticospinal excitability in the muscles which are active when actually performing the observed (AO) or imagined (MI) movements [7,8]. All recorded muscles in the present study are recruited during actual walking. However, the results of the present study showed that tSCS-evoked spinal reflex amplitudes during the AO + MI condition and, compared to the control condition, significantly increased in the SOL muscle and showed the same tendency in the TA and MG muscles but were not influenced in the VM and BF muscles (Table 1, Figure 3). Moreover, correlation analyses indicated that the modulation patterns of the spinal reflexes were similar between lower-leg muscles (TA, SOL, and MG) and between thigh muscles (VM and BF) during AO + MI (Table 3, Figure 4b). Thus, these results suggested muscle-specific facilitation of spinal reflexes during AO + MI unrelated to muscle activity during actual walking. Previous studies showed an increase in the H-reflex during AO and MI, suggesting a cortical output generated during AO and MI affecting the sites located below the cerebral cortex such as the spinal cord, thus modulating spinal reflexes [5,6,9,13]. In the present study, participants were asked to maintain the supine position and perform each experimental task without action execution. Consequently, there were no background EMG signals in each condition and no significant differences among the background EMG activities of any muscle under any condition. Thus, possible effects of both somatosensory inputs and motor commands from higher supraspinal centers on the spinal reflex amplitudes can be excluded. In line with the previous studies, the activity of cortical areas related to AO and MI may facilitate the excitability of the spinal motoneurons at a subthreshold level. 

Interestingly, AO + MI increased in our results similarly the spinal reflex amplitudes only in the distal leg muscles that are farther away from the brain generating the cortical output compared to the thigh muscles in which the spinal reflex amplitudes did not change (Table 3, Figure 4b). If the facilitatory effects of AO + MI on spinal reflexes were dependent on the spinal segment level, the modulation patterns of the spinal reflexes would be similar between muscles that have the same innervation. Although the SOL, MG, and BF muscles are innervated from sacral segments of the spinal cord, AO + MI facilitated spinal reflexes only in the SOL and MG muscles but not in the BF muscle. These results indicated that the facilitatory effects of AO + MI on spinal reflexes depend on the muscles rather than the spinal segments. The stimulus intensity was set to induce spinal reflexes on the ascending limb of the recruitment curve for all muscles (Figure 2a). Thus, the muscle-specific facilitation of spinal reflexes during AO + MI was not due to ceiling or floor effects of the spinal reflex amplitudes but suggested a difference in sensitivity to cortical output generated by AO and/or MI of walking among lower-limb muscles. 

A previous study using tSCS investigated the modulation of spinal reflexes in the lower-limb muscles during MI of finger pinching and ankle plantar flexion [21]. This study showed that the MI of both pinching and plantar flexion increased spinal reflex amplitudes in plantar and dorsal flexor muscles but not in thigh and toe muscles, indicating muscle-specific facilitation of spinal reflexes during MI. Taken together, it is suggested that the lower-leg muscles are more sensitive to MI effects on spinal reflexes than thigh muscles. Differences in the modulation of spinal reflexes between lower-limb muscles during AO + MI and MI are thought to be caused by variations in connectivity strength between the sensorimotor area and the specific muscle. Another study examined electroencephalogram (EEG)–EMG coherence while maintaining a constant force level at 30% MVC of four lower-limb muscles. This study demonstrated that the magnitude of the EEG–EMG coherence was significantly greater in the TA and SOL muscles than in the BF and rectus femoris muscles [22]. Furthermore, a study investigated the corticomuscular connectivity, including information flow direction during walking. The results indicated that the connectivity of the motor cortex was stronger to the TA muscle than to the BF and VM muscles [23]. Therefore, the increased connectivity between the sensorimotor cortex and lower-leg muscles during voluntary contraction or walking might explain that the muscle-specific facilitation of spinal reflexes during AO + MI is increased in TA, SOL, and MG muscles but not in VM and BF muscles (Table 3, Figure 4b). 

### 4.2. Slight Facilitation of Spinal Reflexes in TA and BF Muscles during AO

The results of the present study showed that, compared to the control condition, tSCS-evoked spinal reflex amplitudes during the AO condition tended to be increased in TA and BF muscles but not in SOL, MG, and VM muscles (Table 1, Figure 3). For the first time, the present study used tSCS to examine AO effects on spinal reflexes in the lower-limb muscles other than the SOL muscle and showed that AO facilitated spinal reflexes in TA and BF muscles but had only minor effects. However, this facilitation of spinal reflexes during AO did not occur in SOL, MG, and VM muscles (Table 1, Figure 3). Previous studies using TMS have reported that MEP facilitation during AO occurs in muscles related to the observed movement [7,24,25]. During walking, the TA and BF muscles are mainly active in the swing phase whereas the SOL, MG, and VM muscles are primarily activated in the stance phase. A published study indicated that the connectivity of the motor cortex with the muscles was stronger in the swing phase than in the stance phase [23]. Furthermore, this study showed that the connectivity of the motor cortex to the TA and BF muscles was stronger than that to the VM muscle. Thus, it was speculated that AO of walking increased spinal reflexes in the TA and BF muscles recruited during the swing phase in which the connectivity between motor cortex and lower-limb muscles is strong during actual walking. Therefore, it was suggested that the muscle-specific facilitation of spinal reflexes during AO is related to the connectivity between motor cortex and lower-limb muscles during actual walking, as well as the facilitation during AO + MI. 

### 4.3. Facilitation of MEPs and Spinal Reflexes in TA and SOL Muscles during AO + MI

Our previous and present studies showed that both MEP [12] and spinal reflex (Table 1) amplitudes in the TA and SOL muscles during AO + MI were increased compared to those during the control condition, regardless of the observed walking phase. These results indicated that AO + MI of walking affects not only the excitability of the corticospinal tract but also that of spinal reflex circuits. Similar modulation patterns of MEPs and spinal reflexes during AO + MI in the TA and SOL muscles have been suggested to be related to changes in the central nervous system during actual walking and ankle dorsal and plantar flexion movements. While walking, the corticospinal excitability of the TA muscle increases not only in the swing phase when this muscle is active, but also in the stance phase when the SOL muscle is active [26]. Trinastic et al. previously examined the brain activity during ankle dorsal and plantar flexion movements. According to their study, a partly common neural mechanism controls ankle dorsal and plantar flexion movements [27]. This suggests that an interaction and/or overlap of neural mechanisms related to the activation of TA and SOL muscles would cause similar modulations of corticospinal excitability in those muscles during AO + MI of walking. In the present study, there is an antagonistic relationship between groups of muscles with similar modulation patterns of spinal reflexes during AO + MI (i.e., between TA, SOL, and MG muscles and VM and BF muscles; Table 3, Figure 4b). During actual walking, spinal reflex modulations are significantly correlated between TA and SOL muscles, as well as between VM and BF muscles, and these two muscle groups act antagonistically [16]. Thus, the similar modulation patterns of spinal reflexes during AO + MI may reflect those during walking. 

### 4.4. Difference in Modulation between H-Reflex and tSCS during AO

Our previous study examined H-reflex modulations during AO and AO + MI of walking [13] and revealed in the SOL muscle similar and contradictory modulations of H-reflex and tSCS-evoked spinal reflex. AO + MI facilitated the H-reflex and tSCS-evoked spinal reflex similarly regardless of the observed phase, whereas AO did not facilitate either reflex but phase-dependently modulated the H-reflex ([13], Figure 2). In the previous study, the participants were asked to sit in a chair during H-reflex measurements, whereas participants of the current study were asked to maintain the supine position during tSCS measurements. When applying tSCS, the supine position is mostly recommended over the sitting position because contractions in trunk muscles affect the stimulus efficiency [28]. The observed discrepancies in modulations of spinal reflex excitability during AO may be caused by posture differences during the measurements. It has been reported that both posture and hip joint angle affect spinal inhibitory interneurons; presynaptic inhibition is suppressed in the supine position and at a hip joint angle of around 0 degrees [29,30]. Therefore, differences in posture and hip joint angle were thought to cause the noted study differences regarding modulations of H-reflex and tSCS-evoked spinal reflex in the SOL muscle during AO. The tSCS-evoked spinal reflexes during AO were not phase-dependently modulated because the supine position may have suppressed the spinal inhibitory mechanisms, which in turn facilitated these reflexes. 

## 5. Conclusions

Using tSCS, our study examined the effects of AO and AO + MI of walking on spinal reflexes of lower-limb muscles, resulting in the facilitation of spinal reflexes in TA and BF muscles during AO, as well as in TA, SOL, and MG muscles during AO + MI. Furthermore, during AO + MI, similar modulation patterns were observed in antagonistic muscle groups. These results demonstrated the muscle-specific facilitation of spinal reflexes during AO and AO + MI and suggested that this facilitation depends on the connectivity strength between the sensorimotor area and the muscle during the actual movement. These findings have elucidated the underlying neural activities induced by AO, MI, and their combined processes and may yield clinically useful information to lead to better neurorehabilitation strategies for patients with neurological gait disorders.

## Figures and Tables

**Table 1:** Summary of the statistical analysis (one-sample *t*-test) comparing transcutaneous spinal cord stimulation (tSCS)-evoked reflexes between action observation (AO) with or without motor imagery (MI) and control conditions

Muscle	Condition	Mean ± SEM	*t* Value	*p* Value	Effect Size
		(%)			d
TA	AO	103.7 ± 1.9	*t* (10) = 1.99	0.078 ^†^	0.827
	AO + MI	106.7 ± 3.3	*t* (10) = 2.04	0.072 ^†^	0.844
SOL	AO	102.9 ± 2.5	*t* (10) = 1.13	0.288	0.5
	AO + MI	105.6 ± 2.2	*t* (10) = 2.51	0.034 *	0.991
MG	AO	103.0 ± 2.4	*t* (10) = 1.27	0.235	0.56
	AO + MI	106.5 ± 3.2	*t* (10) = 2.03	0.073 ^†^	0.842
VM	AO	100.3 ± 2.1	*t* (10) = 0.147	0.887	0.067
	AO + MI	100.1 ± 4.5	*t* (10) = 0.037	0.971	0.017
BF	AO	104.3 ± 2.2	*t* (10) = 1.98	0.079 ^†^	0.825
	AO + MI	101.1 ± 4.3	*t* (10) = 0.246	0.811	0.113

^†^*p* < 0.1, * *p* < 0.05. TA, tibialis anterior muscle; SOL, soleus muscle; MG, medial gastrocnemius muscle; VM, vastus medialis muscle; BF, biceps femoris long head muscle.

**Table 2:** Summary of statistical analyses comparing transcutaneous spinal cord stimulation (tSCS)-evoked reflexes between action observation (AO) and AO with motor imagery (AO + MI) conditions and among the four phases in each condition using repeated-measures (rm)-ANOVAs.

Muscle	Method		*F* Value	*p* Value	Effect Size η_p_^2^
TA	two-way rm-ANOVA one-way rm-ANOVA	Condition	F (1,9) = 1.290	0.285	0.125
		Phase	F (3, 27) = 0.094	0.766	0.010
		Condition × Phase	F (3,27) = 0.573	0.659	0.068
		Phase (AO)	F (3,27) = 1.160	0.343	0.114
		Phase (AO + MI)	F (3,27) = 0.106	0.956	0.012
SOL	two-way rm-ANOVA one-way rm-ANOVA	Condition	F (1,9) = 1.400	0.267	0.134
		Phase	F (3,27) = 0.015	0.904	0.002
		Condition × Phase	F (3,27) = 0.340	0.574	0.036
		Phase (AO)	F (3,27) = 0.430	0.733	0.046
		Phase (AO + MI)	F (3,27) = 0.144	0.933	0.016
MG	two-way rm-ANOVA one-way rm-ANOVA	Condition	F (1,9) = 1.430	0.262	0.137
		Phase	F (3,27) = 0.731	0.415	0.075
		Condition × Phase	F (3,27) = 0.211	0.657	0.023
		Phase (AO)	F (3,27) = 1.310	0.291	0.127
		Phase (AO + MI)	F (3,27) = 0.294	0.829	0.032
VM	two-way rm-ANOVA one-way rm-ANOVA	Condition	F (1,9) = 0.006	0.938	0.001
		Phase	F (3,27) = 0.544	0.656	0.057
		Condition × Phase	F (3,27) = 1.220	0.321	0.120
		Phase (AO)	F (3,27) = 0.981	0.416	0.098
		Phase (AO + MI)	F (3,27) = 0.670	0.578	0.069
BF	two-way rm-ANOVA one-way rm-ANOVA	Condition	F (1,9) = 0.770	0.403	0.079
		Phase	F (3,27) = 0.032	0.861	0.004
		Condition × Phase	F (3,27) = 2.060	0.185	0.186
		Phase (AO)	F (3,27) = 0.258	0.855	0.028
		Phase (AO + MI)	F (3,27) = 0.997	0.409	0.100

TA, tibialis anterior muscle; SOL, soleus muscle; MG, medial gastrocnemius muscle; VM, vastus medialis muscle; BF, biceps femoris long head muscle.

**Table 3:** Pearson correlation coefficients (*r*) and *p* values (*p*) of transcutaneous spinal cord stimulation (tSCS)-evoked spinal reflex amplitudes (% of control) between pairs of lower-limb muscles in action observation (AO) and AO with motor imagery (AO + MI) conditions

Condition	Muscle	TA	SOL	MG	VM
AO	SOL	*r* = 0.973 *p* < 0.001 *			
	MG	*r* = 0.713 *p* = 0.021 *	*r* = 0.800 *p* = 0.005 *		
	VM	*r* = 0.635 *p* = 0.048 *	*r* = 0.775 *p* = 0.009 *	*r* = 0.704 *p* = 0.023 *	
	BF	*r* = 0.396 *p* = 0.258	*r* = 0.464 *p* = 0.176	*r* = 0.156 *p* = 0.688	*r* = 0.551 *p* = 0.099 ^†^
AO + MI	SOL	*r* = 0.876 *p* < 0.001 *			
	MG	*r* = 0.911 *p* < 0.001 *	*r* = 0.913 *p* < 0.001 *		
	VM	*r* = 0.418 *p* = 0.230	*r* = 0.452 *p* = 0.190	*r* = 0.442 *p* = 0.201	
	BF	*r* = 0.295 *p* = 0.408	*r* = 0.424 *p* = 0.222	*r* = 0.361 *p* = 0.306	*r* = 0.956 *p* < 0.001 *

^†^*p* < 0.1, * *p* < 0.05. TA, tibialis anterior muscle; SOL, soleus muscle; MG, medial gastrocnemius muscle; VM, vastus medialis muscle; BF, biceps femoris long head muscle.
